# Associations of Dietary Zinc Supplementation and Sleep Patterns with Chronic Kidney Disease Risk: A Prospective Cohort Study

**DOI:** 10.3390/healthcare13070703

**Published:** 2025-03-23

**Authors:** Xiaofeng Zhang, Shuai Zhang, Jiali Lv, Xiaoyan Ma, Xia Lin, Lin Yang, Shengxu Li, Tao Zhang

**Affiliations:** 1 Department of Biostatistics, School of Public Health, Cheeloo College of Medicine, Shandong University, Jinan 250012, China; 202216394@mail.sdu.edu.cn (X.Z.); zhangshuai522@mail.sdu.edu.cn (S.Z.); jialilv@mail.sdu.edu.cn (J.L.); 202216373@mail.sdu.edu.cn (X.M.); 202236450@mail.sdu.edu.cn (X.L.); 2 Institute for Medical Dataology, Cheeloo College of Medicine, Shandong University, Jinan 250012, China; 3 Department of Cancer Epidemiology and Prevention Research, Cancer Research & Analytics, Cancer Care Alberta, Alberta Health Services, Calgary, AB T2V 0N5, Canada; 4 Departments of Oncology and Community Health Sciences, Cumming School of Medicine, University of Calgary, Calgary, AB T2V 0N5, Canada; 5 Children’s Minnesota Research Institute, Children’s Minnesota, Minneapolis, MN 55404, USA; lishx@hotmail.com

**Keywords:** chronic kidney disease, dietary zinc supplementation, sleep patterns, zinc–sleep interaction, prospective cohort study

## Abstract

Background: Previous studies have indicated that both dietary zinc supplementation and sleep patterns may influence the development of chronic kidney disease (CKD). Additionally, it is established that dietary zinc can enhance sleep quality. Despite these insights, the interplay between zinc supplementation and sleep patterns, and their combined effect on CKD progression, is still not fully understood. Methods: This population-based cohort study used UK Biobank data (2006–2010) and employed cox regression models to assess the associations between dietary zinc supplementation, sleep patterns, and their combined effects on CKD. Results: Over a median follow-up of 14.8 years, 22,384 new CKD cases were identified. Zinc supplementation reduced CKD risk in individuals with poor (HR: 0.70, 95% CI: 0.50–0.98) and moderate (HR: 0.89, 95% CI: 0.81–0.98) sleep patterns but not in those with healthy sleep (HR: 1.00, 95% CI: 0.89–1.14). A significant interaction between zinc supplementation and sleep patterns was observed (*p* = 0.017), with sensitivity analyses confirming the results. Conclusions: These findings indicate a significant association between dietary zinc supplementation and reduced CKD risk, especially in individuals with poor sleep patterns. Further studies are needed to explore zinc supplementation as a targeted intervention for those at higher CKD risk due to poor sleep.

## 1. Introduction

Chronic kidney disease (CKD) has become a major global health concern, with its prevalence rising by 29.3% since 1990 and its mortality rate increasing by 41.5% from 1990 to 2017 [1,2]. Currently, CKD affects over 800 million individuals worldwide and is projected to become the fifth leading cause of years of life lost by 2040 [3]. This alarming trend highlights the urgent need for innovative prevention and management strategies to reduce the growing global health burden of CKD.

Zinc, an essential trace element, plays pivotal roles in catalytic, structural, and regulatory processes [4]. It also acts as an antioxidant with anti-inflammatory properties and is crucial in regulating both innate and adaptive immune responses, enhancing resistance to infections [5]. Numerous studies have established a link between zinc supplementation and various health conditions, including CKD [6,7,8].

In addition to nutritional factors, sleep patterns, such as sleep duration and snoring, are vital for kidney health. A growing body of evidence, including epidemiological studies and meta-analyses, has linked sleep dysfunctions, such as abnormal sleep duration, insomnia, snoring, and daytime dysfunction, with the development and progression of CKD [9,10,11,12,13,14,15]. Interestingly, zinc is also implicated in sleep regulation, as it contributes to the synthesis and metabolism of key neurotransmitters such as serotonin and melatonin, which are essential for sleep modulation [16]. Moreover, zinc influences sleep–wake cycles by modulating gamma-aminobutyric acid (GABA) and glutamate receptor activity in the brain [17]. These dual roles suggest that both dietary zinc intake and sleep patterns should be considered in CKD prevention strategies.

Although several studies have explored the effects of zinc supplementation on sleep [18,19,20], the interactive effects of dietary zinc supplementation and sleep patterns on CKD risk remain underexplored. To address this gap, the present study leverages the extensive and well-characterized UK Biobank cohort to investigate how dietary zinc supplementation influences CKD risk and whether this relationship is modified by sleep patterns.

## 2. Methods

### 2.1. Study Design and Participants

UK Biobank is a large-scale prospective cohort study that enrolled over 500,000 participants, aged 37–73 years, from the general population across the United Kingdom between 2006 and 2010. Designed as a long-term prospective study with planned follow-up until 2036, it aims to longitudinally track participants’ health and medical conditions. Upon enrollment, participants provided electronic consent. Data collection included touchscreen questionnaires, physical measurements, and biological samples, all conducted at 22 assessment centers across the United Kingdom. Written informed consent was obtained prior to any data collection, ensuring compliance with ethical standards.

**Figure 1:**
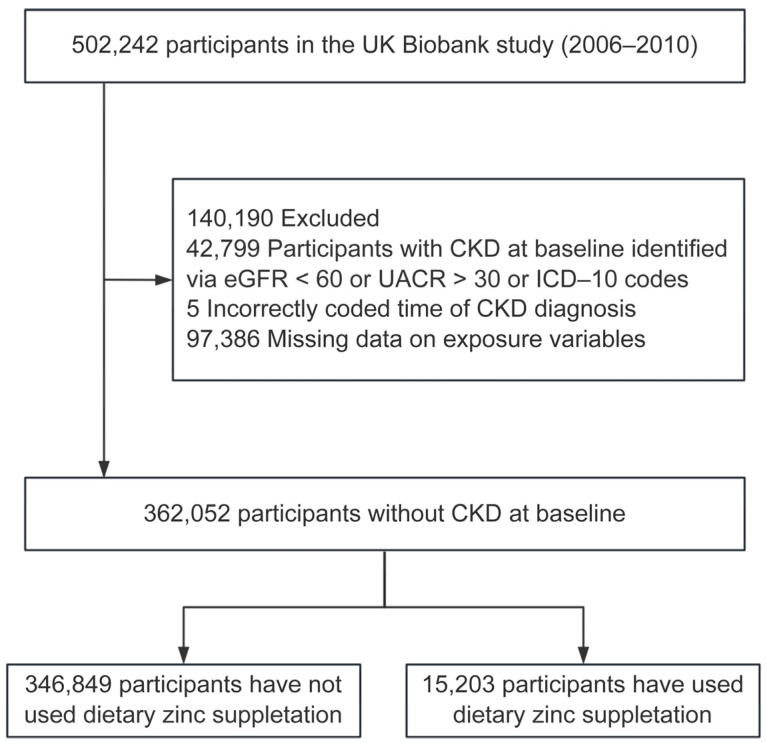
Flowchart of the participant inclusion in the study. Abbreviations: CKD = chronic kidney disease; eGFR = estimated glomerular filtration rate; UACR = urinary albumin–creatinine ratio; ICD-10 = the international statistical classification of diseases and related health problems 10th revision.

In the initial cohort of 502,242 participants, 42,799 were excluded due to prevalent CKD at baseline, and 97,386 were excluded due to missing data on dietary zinc supplementation, sleep patterns, and other relevant variables. The final cohort comprised 362,052 participants, of whom 15,203 reported using dietary zinc supplementation, while 346,849 did not. Figure 1 illustrates the inclusion and exclusion criteria applied during participant selection.

### 2.2. Primary Exposure

During the baseline assessment, participants completed a touchscreen questionnaire that assessed their habitual supplement intake. One of the questions asked “Do you regularly take any of the following?” Participants could indicate their use of zinc supplementation among the options provided. Habitual zinc use was categorized as follows: 0 = no, 1 = yes.

The questionnaire also collected comprehensive data on various aspects of sleep behavior, structured as follows: (1) Sleep duration was assessed with the question “About how many hours of sleep do you get in every 24 h, including naps?” (2) Chronotype was determined by the question “Do you consider yourself to be?” Options included “definitely a morning person”, “definitely an evening person”, “do not know”, and “prefer not to answer”. (3) Insomnia was evaluated using the question “Do you have trouble falling asleep at night or do you wake up in the middle of the night?” Responses ranged from “never/rarely” to “usually”. (4) Snoring was assessed with the question “Does your partner or a close relative or friend complain about your snoring?” Response options included “yes”, “no”, “don’t know”, and “prefer not to answer”. (5) Daytime sleepiness was assessed with the question “How likely are you to doze off or fall asleep during the daytime when you do not mean to?” Responses ranged from “never/rarely” to “all the time”. Participants were instructed to provide an average response for the past month if their sleep behaviors varied. Participants who selected “Do not know” or “Prefer not to answer” were excluded from the analysis (Supplementary Table S1).

Low-risk sleep behaviors were defined based on specific criteria: (1) sleep duration of 7 to 8 h per day; (2) an early chronotype, defined as either “morning” or “more morning than evening”; (3) absence of reported snoring; (4) infrequent insomnia episodes, rated as “never/rarely” or “sometimes”; and (5) minimal daytime sleepiness, described as “never/rarely” or “sometimes”. Participants received 1 point for each low-risk behavior exhibited and 0 points if not. The total healthy sleep score was calculated by summing the points from all five sleep behaviors, with higher scores indicating healthier sleep patterns.

Based on these criteria, participants were classified into three sleep quality groups: scores of 0–1 as the “poor sleep” group, scores of 2–3 as the “moderate sleep” group, and scores of 4–5 as the “healthy sleep” group [21]. This classification enables a comprehensive analysis of how sleep patterns influence health outcomes.

### 2.3. Ascertainment of CKD

Incident CKD cases were identified using ICD codes (N03, N06, N08, N11, N12, N13, N14, N15, N16, N18, N19, N20, and N21) recorded in primary care data, hospital inpatient data, and death register records [21]. Data from hospital inpatients were obtained through linkage with the Hospital Episode Statistics in England, the Scottish Morbidity Records in Scotland, and the Patient Episode Database in Wales.

Prevalent CKD was defined as an estimated glomerular filtration rate (eGFR) below 60 mL/min per 1.73 m^2^ at recruitment, a urinary albumin–creatinine ratio (UACR) greater than 30 mg/g at baseline, or identification using the aforementioned ICD-10 codes [22]. Further details on CKD case identification are provided in Supplementary Table S2.

### 2.4. Assessment of Other Covariates

At baseline, data on demographic and socioeconomic factors, lifestyle behaviors, medical history, and medication use were collected through touchscreen questionnaires and nurse-led interviews. Body mass index (BMI) was calculated from measured height and weight, expressed in kg/m^2^. Racial categories were classified as “White” or “Non-White”. Educational attainment was categorized into four groups: “Higher degree”, “Any school degree”, “Vocational qualifications”, and “None of the above”. Smoking and drinking statuses were also categorized as “Never”, “Previous”, or “Current”.

Vitamin supplementation was defined as the intake of any of the following: vitamin A, B (including folic acid or folate), C, D, or E and was recorded as “Yes” or “No”. Similarly, other mineral supplementation was defined as the intake of any of the following: fish oil, glucosamine, calcium, iron, or selenium and was recorded as “Yes” or “No”.

Hypertension was defined as a measured blood pressure of ≥140/90 mmHg, a self-reported physician diagnosis, or the use of antihypertensive medications. Diabetes was identified through self-reported physician diagnosis, insulin use, or an HbA1c level of 6.5% or higher. Physical activity levels were assessed using the self-reported short-form International Physical Activity Questionnaire, with results summarized in MET minutes per week and classified into low, moderate, or high activity levels.

### 2.5. Statistical Analysis

The Anderson–Darling goodness-of-fit test was employed in this study to perform the normality hypothesis test [23]. Participant characteristics are presented as means ± SD for normally distributed continuous variables, medians with interquartile ranges (IQR) for non-normally distributed continuous variables, and frequencies with percentages for categorical variables. The follow-up period began on the baseline date, defined as the date of initial attendance at the assessment center, and continued until either the diagnosis of CKD or the censoring date (31 December 2023), whichever occurred first.

We employed Cox proportional hazards regression models to evaluate the associations between zinc supplementation, sleep patterns, and the risk of incident CKD. The Schoenfeld residuals method was used to test the proportional hazards assumption of the Cox models, and no violations were detected [24]. In Model 1, adjustments were made for age and sex. Model 2 included additional adjustments for race, BMI, education level, Townsend deprivation index (TDI), drinking status, smoking status, physical activity, vitamins and other mineral supplementations, and dietary habits. Model 3, the full model, also included adjustments for hypertension and diabetes. Missing data were handled by coding them into a separate missing indicator category. Details regarding the number of missing covariates are provided in Supplementary Table S3.

To explore whether sleep patterns modify the association between zinc supplementation and CKD risk, interaction terms on a multiplicative scale were evaluated. The joint associations were analyzed by stratifying participants into six groups based on their habitual use of zinc supplements (non-users or users) and sleep patterns (poor, moderate, or healthy). HRs for incident CKD were calculated across these groups, and cumulative incidence curves were constructed for comparison with those of non-supplement users.

Stratified analyses were conducted across various demographics and health characteristics to separately evaluate the effects of zinc supplementation and sleep patterns, including age (<65 or ≥65 years); sex (male or female); education level (higher degree, school degree, or vocational qualifications); ethnic background (Non-White or White); BMI categories (underweight, normal weight, overweight, or obese); hypertension (yes or no); and diabetes status (yes or no).

A series of sensitivity analyses was conducted to assess the robustness of our findings. To mitigate the potential effects of reverse causation, participants who developed CKD within the first two years of follow-up were excluded from the analysis. Additionally, the main analyses were repeated in a 1:4 propensity score-matched cohort to reduce confounding. Lastly, we focused on participants with the CKD diagnosis code N18, as N18 represents the most commonly diagnosed CKD code.

All analyses were performed using R software, version 4.2.1. A two-sided *p*-value of less than 0.05 was considered statistically significant.

This study follows the STROBE guidelines for reporting observational studies, as recommended by the EQUATOR Network [25].

## 3. Results

### 3.1. Baseline Characteristics

The baseline characteristics of the study population were analyzed based on dietary zinc supplementation use (Table 1). Of the 362,052 participants, the average age was 56.2 years (SD = 8.1), and 55.6% were female. A total of 15,203 (4.2%) reported the use of dietary zinc supplementation. Users were typically wealthier, predominantly female, possessed higher educational levels, had a lower BMI, and showed a reduced prevalence of type 2 diabetes and hypertension. They also engaged in higher levels of physical activity, adhered to healthier diets, and were less likely to smoke or consume alcohol. Over a median follow-up period of 14.8 years (IQR, 14.1–15.6 years), 22,384 new cases of CKD (6.2%) were identified.

### 3.2. Dietary Zinc Supplementation, Sleep Patterns, and CKD

The results of the study indicate that the use of dietary zinc supplementation and adherence to healthy sleep patterns are associated with a reduced risk of CKD (Table 2). HRs indicate a notable reduction in CKD risk, ranging from 0.84 (95% CI: 0.78–0.90) in Model 1 to 0.92 (95% CI: 0.85–0.99, *p* = 0.026) in Model 3. Moreover, healthier sleep patterns were significantly associated with a lower risk of CKD, with HRs of 0.61 (95% CI: 0.58–0.65) for healthy sleep and 0.73 (95% CI: 0.69–0.77) for moderate sleep in Model 1, and these associations remained significant in the subsequent models. An inverse trend in CKD risk was consistently observed with improving sleep patterns (*p* < 0.001).

**Figure 2:**
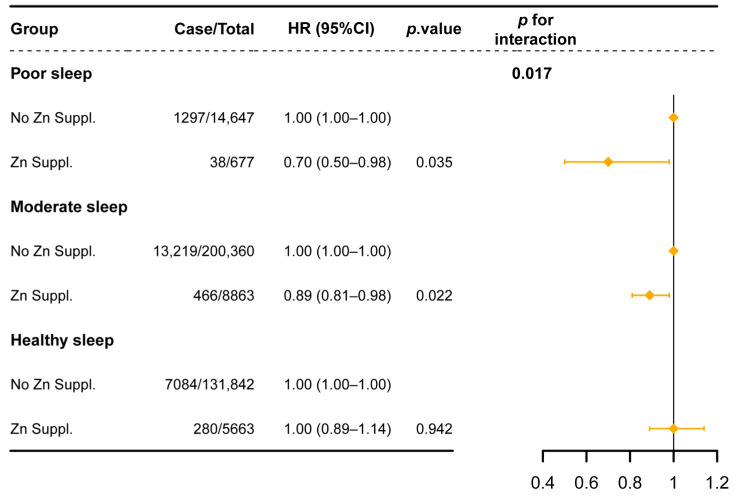
Association between dietary zinc supplementation and CKD incidence across different sleep patterns. Notes: HRs (95% CI) were adjusted for age (continuous); sex (male or female); race (White or Non-White); BMI (underweight, normal weight, overweight, or obese); education level (higher degree, any school degree, vocational qualifications, or none of the above); TDI; drinking status (never drinking, former drinking, or current drinking); smoking status (never smoker, former smoker, or current smoker); physical activity (low, moderate, or high); vitamin supplementation (yes or no); other mineral supplementation (yes or no); diet score; hypertension (yes or no); and diabetes (yes or no). Abbreviations: Zn Suppl. = zinc supplementation.

The effect of dietary zinc supplementation on the reduction of CKD incidence risk varied across different sleep pattern groups (Figure 2). The reduced risk of CKD associated with zinc supplementation progressively diminished as sleep patterns transitioned from “poor” to “healthy” (HR = 0.70, 95% CI 0.50–0.98, *p* = 0.035 in those with a poor sleep pattern; HR = 0.89, 95% CI 0.81–0.98, *p* = 0.022 in those with a moderate sleep pattern; and HR = 1.00, 95% CI 0.89–1.14, *p* > 0.05 in those with a healthy sleep pattern). The interaction effect between zinc supplementation and sleep patterns on CKD risk was significant (*p* for interaction = 0.017).

**Figure 3:**
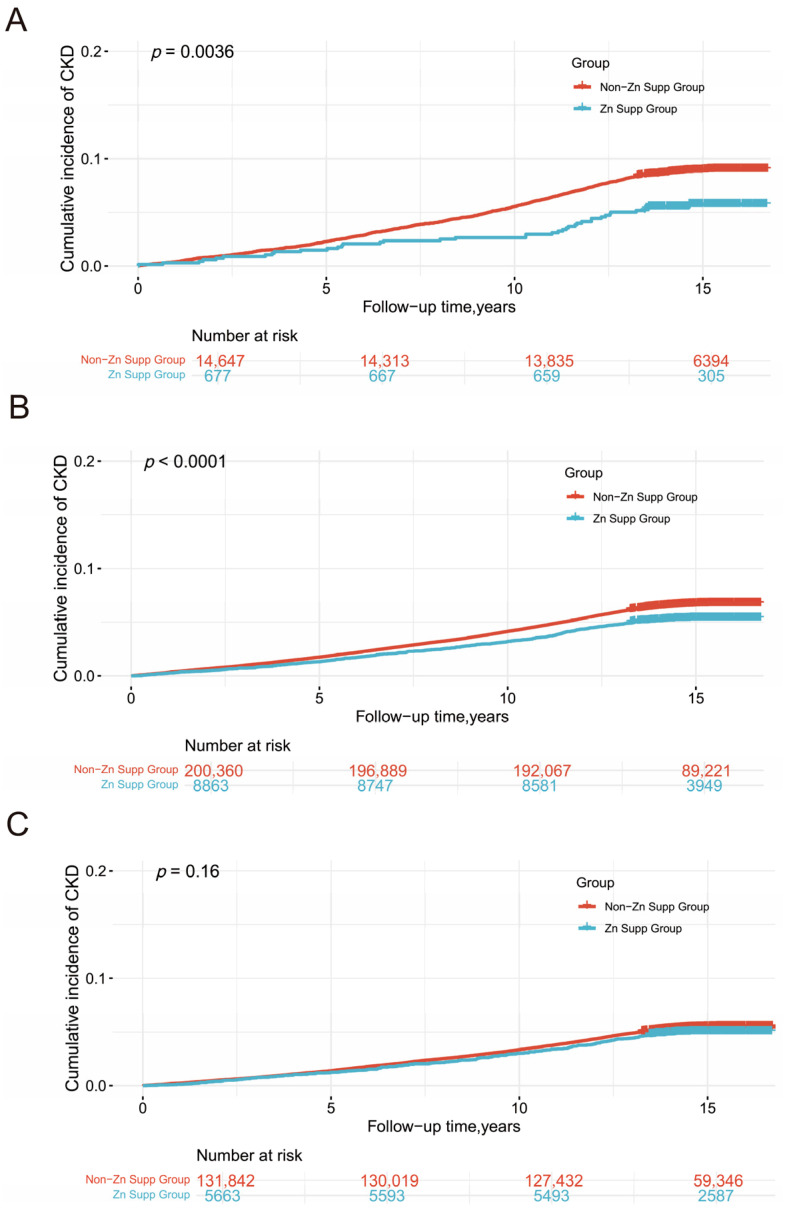
Cumulative incidence of CKD by dietary zinc supplementation in individuals with different sleep patterns. Notes: (**A**) Cumulative incidence of CKD among individuals with poor sleep patterns, stratified by dietary zinc supplementation use. (**B**) Cumulative incidence of CKD among individuals with moderate sleep patterns, stratified by dietary zinc supplementation use. (**C**) Cumulative incidence of CKD among individuals with healthy sleep patterns, stratified by dietary zinc supplementation use. Abbreviations: Non-Zn Supp Group: no dietary zinc supplementation. Zn Supp Group: with dietary zinc supplementation.

The 10-year cumulative incidence of CKD was assessed based on zinc supplementation status, stratified by sleep pattern (Figure 3). Among individuals with poor sleep patterns, those receiving zinc supplementation showed a significantly lower incidence of CKD than those without supplementation (*p* = 0.0036). Similarly, in the moderate sleep group, zinc supplementation was associated with a significant reduction in CKD incidence (*p* < 0.0001). However, in the healthy sleep group, no significant difference in CKD incidence was found between those with and without zinc supplementation (*p* = 0.16). Additionally, we present the 10-year cumulative incidence curves for CKD across the three sleep pattern groups, independent of zinc supplementation (Supplementary Figure S1).

The relationship between dietary zinc supplementation and CKD risk was examined, with segmentation based on individual sleep behavior status across five sleep-related variables: sleep duration, chronotype, insomnia, snoring, and daytime sleepiness (Table 3). The association between zinc supplementation and CKD risk was generally stronger in higher-risk groups, with a statistically significant interaction observed only for snoring.

### 3.3. Subgroup and Sensitivity Analyses

No significant interactions were found between dietary zinc supplementation and the subgroups defined by age, sex, educational level, race, BMI, hypertension, or diabetes. However, the association between CKD risk and healthy sleep patterns was stronger in those younger than 65 years compared to older individuals and in non-diabetic patients compared to diabetic participants, with interaction *p*-values of <0.001 and *p* = 0.032, respectively. (Supplemental Table S4)

Extensive sensitivity analyses were conducted. These analyses included the exclusion of cases diagnosed within the first two years of cohort entry (Supplemental Tables S5 and S6), analysis within a 1:4 propensity score-matched cohort (Supplemental Tables S7 and S8), and analysis restricted to individuals with a primary CKD diagnosis code of N18 (Supplemental Tables S9 and S10). Overall, the results remained consistent across these sensitivity analyses.

## 4. Discussion

In this large prospective cohort study involving individuals without pre-existing CKD, dietary zinc supplementation and healthy sleep patterns were associated with a lower risk of CKD. Notably, a significant interaction was observed between dietary zinc supplementation and sleep patterns. The protective effect of zinc supplementation was particularly pronounced among individuals with unhealthy sleep patterns, suggesting a potential role for zinc in mitigating CKD risk among individuals with poor sleep patterns.

Our findings align with previous studies suggesting that zinc supplementation and healthy sleep patterns may help prevent various diseases [26,27,28,29,30]. For instance, data from the Korean Genome and Epidemiology Study, a prospective community-based cohort study, suggest that inadequate dietary zinc intake elevates CKD risk in individuals with normal renal function [31]. A cross-sectional analysis of NHANES data (2003–2018) indicates that higher dietary zinc intake is significantly associated with a reduced risk of CKD [32]. Furthermore, prospective cohort studies using data from UK Biobank [11,21,33] consistently demonstrate an inverse association between adherence to healthy sleep behaviors and CKD risk. Our findings align with these prior studies and support existing guideline recommendations emphasizing the critical role of healthy sleep in CKD prevention. Additionally, these guidelines underscore the need for further investigation into nutritional interventions, including zinc supplementation, to strengthen the evidence base regarding their potential effectiveness in reducing CKD risk [34].

To our knowledge, this study is the first to demonstrate the link between dietary zinc supplementation and CKD risk within a large prospective cohort. It is also the first study to investigate the interaction between dietary zinc supplementation and sleep patterns on CKD risk, providing novel insights into the development of CKD.

Potential explanations for the protective effects of dietary zinc supplementation in reducing the risk of CKD include (1) zinc’s crucial role in diminishing oxidative stress levels, which is widely acknowledged as a significant factor contributing to the progression of renal diseases [35,36,37,38,39]. (2) Additionally, zinc’s anti-fibrotic effects may prevent morphological changes in the kidneys, thereby reducing the incidence of kidney diseases [40,41].

The interaction between zinc and sleep primarily involves zinc’s modulatory effects in the central nervous system, where it inhibits the release of the excitatory neurotransmitter glutamate [42]. This inhibition may contribute to improved sleep quality. Adequate sleep is critical for regulating the pathophysiological processes associated with CKD, as research has demonstrated that adequate sleep can suppress inflammatory pathways triggered by poor sleep quality, notably by reducing the accumulation of pro-inflammatory cytokines such as interleukin-1β, interleukin-6, and tumor necrosis factor α, all of which are significant contributors to endothelial dysfunction [43,44,45]. Additionally, enhancing sleep quality through zinc supplementation may indirectly mitigate key risk factors for CKD, including worsening of obesity, hypertension, diabetes, and vascular diseases [46,47,48,49].

Our study has several notable strengths. First, by utilizing the extensive prospective cohort of UK Biobank, our research uniquely demonstrates how dietary zinc supplementation in conjunction with healthy sleep patterns is associated with a significant reduction in the incidence of CKD. Second, UK Biobank follows rigorous quality assurance protocols, and its data quality is well established. Third, the detailed information available on socioeconomic factors, lifestyle behaviors, and other covariates allows for comprehensive adjustments, minimizing potential confounding influences. Lastly, the large sample size enabled us to conduct extensive subgroup and sensitivity analyses, thereby affirming the robustness of our findings across various population segments and under different analytical conditions.

While our study provides significant insights, several limitations warrant consideration. Firstly, UK Biobank provides only categorical data on the use of zinc supplements and lacks specific information on dosage, formulation, or duration of use. This lack of detailed data hinders a deeper exploration of dose–response relationships and the potential differential effects of various types of zinc formulations or different durations of usage. Second, since exposure information was only collected at baseline, the study does not account for changes in zinc supplementation and sleep patterns over time. Repeated measurements would be necessary to more accurately assess how changes in zinc supplementation and sleep patterns correlate with CKD risk. Thirdly, the observational nature of our study design means that we cannot completely exclude the impact of residual or unknown confounding factors on the results. Thus, this study does not support definitive causal conclusions. Future research should include experimental interventions to validate our findings. Lastly, the demographic profile of our cohort—predominantly middle-aged or older White individuals—may limit the generalizability of our findings to other racial or age groups. Further studies are needed to confirm these associations in different populations.

## 5. Conclusions

In conclusion, this large-scale cohort study demonstrates that dietary zinc supplementation is significantly linked to a lower risk of CKD, especially in individuals with unhealthy sleep patterns. These results highlight zinc’s potential as part of CKD prevention, particularly for those with poor sleep habits.

To validate and expand upon these findings, future randomized controlled trials (RCTs) are essential to systematically evaluate the effects of varying zinc dosages and supplementation durations on CKD prevention in diverse populations with different sleep patterns. Such studies will be crucial in identifying the most effective and personalized intervention strategies [50]. Meanwhile, the integration of cutting-edge technologies such as Artificial Intelligence (AI) and the Internet of Things (IoT) into the nutritional management of chronic diseases holds great promise for innovating CKD prevention approaches and advancing a more proactive and precise health management model [51,52,53].

## Figures and Tables

**Table 1:** Baseline characteristics of the participants from UK Biobank.

Variable	Total	Non-Zn Supp	Zn Supp	*p*-Value
	362,052	346,849	15,203	
Age, years	56.2 (8.1)	56.2 (8.1)	56.2 (8.1)	0.995
TDI	−1.49 (3.0)	−1.49 (3.0)	−1.29 (3.1)	<0.001
Female, *n* (%)	201,201 (55.6)	191,564 (55.2)	9637 (63.4)	<0.001
Education, *n* (%)				<0.001
Higher degree	122,813 (33.9)	116,880 (33.7)	5933 (39.0)	
Any school degree	139,381 (38.5)	133,508 (38.5)	5873 (38.6)	
Vocational qualifications	42,109 (11.6)	40,485 (11.7)	1624 (10.7)	
None of the above	55,271 (15.3)	53,588 (15.4)	1683 (11.1)	
Ethnic white, *n* (%)	331,510 (91.6)	318,106 (91.7)	13,404 (88.2)	<0.001
Diabetes, *n* (%)	17,508 (4.8)	16,881 (4.9)	627 (4.1)	<0.001
BMI, kg/m^2^, *n* (%)				<0.001
underweight	1681 (0.5)	1587 (0.5)	94 (0.6)	
normal weight	119,846 (33.1)	113,945 (32.9)	5901 (38.8)	
overweight	155,289 (42.9)	149,155 (43.0)	6134 (40.3)	
obese	83,669 (23.1)	80,671 (23.3)	2998 (19.7)	
Hypertension, *n* (%)	183,143 (50.6)	176,266 (50.8)	6877 (45.2)	<0.001
Physical activity score, *n* (%)				<0.001
Low	85,155 (23.5)	82,233 (23.7)	2922 (19.2)	
Moderate	136,922 (37.8)	131,327 (37.9)	5595 (36.8)	
High	133,294 (36.8)	126,872 (36.6)	6422 (42.2)	
Smoking, *n* (%)				<0.001
Current	36,421 (10.1)	35,032 (10.1)	1389 (9.14)	
Never	890 (0.3)	850 (0.3)	40 (0.3)	
Previous	199,104 (55.0)	190,926 (55.0)	8178 (53.8)	
Alcohol, *n* (%)				<0.001
Current	336,307 (92.9)	322,330 (92.9)	13,977 (91.9)	
Never	160 (0.04)	152 (0.04)	8 (0.05)	
Previous	13,881 (3.8)	13,319 (3.8)	562 (3.7)	
Healthy diet score	3.5 (1.5)	3.5 (1.5)	4.0 (1.5)	<0.001
Chronotype, *n* (%)				<0.001
“morning” person	96,871 (26.8)	92,932 (26.8)	3939 (25.9)	
“evening” person	31,899 (8.81)	30,297 (8.73)	1602 (10.5)	
More a “morning” than “evening” person	130,208 (36.0)	125,027 (36.0)	5181 (34.1)	
More an “evening” than a “morning” person	103,074 (28.5)	98,593 (28.4)	4481 (29.5)	
Snoring, *n* (%)	133,004 (36.7)	128,030 (36.9)	4974 (32.7)	<0.001
Daytime dozing, *n* (%)				<0.001
All of the time	21 (0.01)	20 (0.01)	1 (0.01)	
Never/rarely	279,172 (77.1)	267,475 (77.1)	11,697 (76.9)	
Often	9160 (2.53)	8742 (2.52)	418 (2.75)	
Sometimes	73,699 (20.4)	70,612 (20.4)	3087 (20.3)	
Sleeplessness, *n* (%)				
Never/rarely	89,644 (24.8)	86,145 (24.8)	3499 (23.0)	<0.001
Sometimes	173,289 (47.9)	165,977 (47.9)	7312 (48.1)	
Usually	99,119 (27.4)	94,727 (27.3)	4392 (28.9)	

Notes: The “Non-Zn Supp Group” includes participants who have not used dietary zinc supplementation. The “Zn Supp Group” includes participants who have used dietary zinc supplementation. Data are present as mean ± SD for continuous variables and n (%) for categorical variables. Abbreviations: TDI = Townsend deprivation index; BMI = body mass index.

**Table 2:** Hazard ratios for the associations of sleep pattern and the use of dietary zinc supplementation with the risk of chronic kidney disease.

Subgroup	Model 1		Model 2		Model 3	
	HR (95%CI)	*p*-Value	HR (95%CI)	*p*-Value	HR (95%CI)	*p*-Value
Zn Suppl.						
No	1.00 (reference)		1.00 (reference)		1.00 (reference)	
Yes	0.84 (0.78–0.90)	<0.001	0.92 (0.84–0.99)	0.019	0.92 (0.85–0.99)	0.026
Sleep pattern						
Poor	1.00 (reference)		1.00 (reference)		1.00 (reference)	
Moderate	0.73 (0.69–0.77)	<0.001	0.83 (0.79–0.88)	<0.001	0.86 (0.81–0.91)	<0.001
Healthy	0.61 (0.58–0.65)	<0.001	0.77 (0.72–0.81)	<0.001	0.80 (0.75–0.85)	<0.001
*p* for trend	-	<0.001	-	<0.001	-	<0.001

Model 1: adjusted for age (continuous) and sex (male or female). Model 2: further adjusted for race (White or Non-White); BMI (underweight, normal weight, overweight, or obese); education level (higher degree, any school degree, vocational qualifications, or none of the above); TDI; drinking status (never drinking, former drinking, or current drinking); smoking status (never smoker, former smoker, or current smoker); physical activity (low, moderate, or high); diet score; vitamin supplementation (yes or no); and other mineral supplementation (yes or no). Model 3: further adjusted for hypertension (yes or no) and diabetes (yes or no). Abbreviations: Zn Suppl. = zinc supplementation; TDI = Townsend deprivation index; BMI = body mass index.

**Table 3:** Hazard ratios for the use of dietary zinc supplements in CKD across different risk strata of sleep behaviors.

Sleep Behaviors	Case Subjects/*N*	HR (95% CI)	*p* for Interaction
Sleep duration			0.068
Low risk	14,204/235,239	0.96 (0.88–1.05)	
High risk	8180/104,429	0.86 (0.75–0.97)	
Chronotype			0.467
Low risk	14,183/212,896	0.95 (0.86–1.04)	
High risk	8201/126,772	0.88 (0.78–0.99)	
Insomnia			0.753
Low risk	4804/84,840	0.88 (0.75–1.03)	
High risk	17,580/254,828	0.93 (0.86–1.01)	
Snoring			0.044
Low risk	13,258/215,790	0.98 (0.89–1.07)	
High risk	9126/123,878	0.83 (0.73–0.94)	
Daytime sleepiness			0.198
Low risk	21,525/331,346	0.93 (0.86–1.00)	
High risk	859/8322	0.69 (0.46–1.05)	

HRs (95% CI) were adjusted for age (continuous); sex (male or female); race (White or Non-White); BMI (underweight, normal weight, overweight, or obese); education level (higher degree, any school degree, vocational qualifications, or none of the above); TDI; drinking status (never drinking, former drinking, or current drinking); smoking status (never smoker, former smoker, or current smoker); physical activity (low, moderate, or high); diet score; vitamin supplementation (yes or no); other mineral supplementation (yes or no); hypertension (yes or no); and diabetes (yes or no).

## Data Availability

All data used in this study are publicly accessible from UK Biobank via their standard data access procedure at https://www.ukbiobank.ac.uk (accessed on 8 February 2024).

## Supplementary Materials

The following supporting information can be downloaded at https://www.mdpi.com/article/10.3390/healthcare13070703/s1: Table S1: The scoring system of sleep behaviors; Table S2: Code lists used in the UK Biobank study to identify CKD; Table S3: The numbers and percentages of participants with missing covariates; Table S4: Subgroup analysis; Table S5: Association between dietary zinc supplementation, sleep patterns, and CKD risk in individuals followed for more than two years; Table S6: Hazard ratios of dietary zinc supplementation and CKD risk stratified by sleep patterns among individuals followed for more than two years; Table S7: Association between dietary zinc supplementation, sleep patterns, and CKD risk among participants with and without CKD in a 1:4 propensity score-matched cohort; Table S8: Hazard ratios of dietary zinc supplementation on CKD risk stratified by sleep pattern using a 1:4 propensity score-matching approach; Table S9: Association between dietary zinc supplementation, sleep patterns, and CKD risk using disease code N18; Table S10: Hazard ratios of dietary zinc supplementation use on CKD risk, stratified by sleep pattern, employing disease code N18 exclusively; Figure S1: Cumulative incidence of CKD stratified by sleep patterns.

## Abbreviations

The following abbreviations are used in this manuscript:

CKD	Chronic kidney disease
TDI	Townsend deprivation index
BMI	Body mass index
HR	Hazard ratio
CI	Confidence interval
eGFR	Estimated glomerular filtration rate
UACR	urinary albumin–creatinine ratio
