# Supplementary material for: Associations of Dietary Zinc Supplementation and Sleep Patterns with Chronic Kidney Disease Risk: A Prospective Cohort Study
Source: Healthcare (Basel). 2025 Mar 23;13(7):703. doi: 10.3390/healthcare13070703 (PMC11988376; doi:10.3390/healthcare13070703)
Supplement: Supplementary file 1 [file healthcare-13-00703-s001.zip › healthcare-3497021-supplementary materials.pdf]

Supplementary materials

Supplemental Table S1. The scoring system of sleep behaviors.

Supplemental Table S2. Code lists used in the UK Biobank study to identify CKD.

Supplemental Table S3. The numbers and percentages of participants with missing covariates.

Supplemental Table S4. Subgroup analysis.

Supplemental Table S5. Association between dietary zinc supplementation, sleep patterns, and CKD risk in individuals followed for more than two years.

Supplemental Table S6. Hazard ratios of dietary zinc supplementation and CKD risk stratified by sleep patterns among individuals followed for more than two years.

Supplemental Table S7. Association between dietary zinc supplementation, sleep patterns, and CKD risk among participants with and without CKD in a 1:4 propensity score matched cohort.

Supplemental Table S8. Hazard ratios of dietary zinc supplementation on CKD risk stratified by sleep pattern using a 1:4 propensity score matching approach.

Supplemental Table S9. Association between dietary zinc supplementation, sleep patterns, and CKD risk using disease code N18.

Supplemental Table S10. Hazard ratios of dietary zinc supplementation use on CKD risk, stratified by sleep pattern, employing disease code N18 exclusively.

Supplemental Figure S1. Cumulative incidence of CKD stratified by sleep patterns.

Table S1. The scoring system of sleep behaviors

Characteristics	Field IDs	UK Biobank Questionnaire	Healthy Answer	Unhealthy Answer
chronotype	1180	Do you consider yourself to be?	Definitely a "morning" person; More a "morning" than "evening" person.	More an "evening" than a "morning person; Definitely an "evening" person
Sleep Duration	1160	About how many hours sleep do you get in every 24 hours? (please include naps)	7-8 hr/d.	<7 or >=9 hr/d
Insomnia	1200	Do you have trouble falling asleep at night or do you wake up in the middle of the night?	Never/rarely	Usually; Sometimes
Snoring	1210	Does your partner or a close relative or friend complain about your snoring?	No	Yes
Daytime Sleepiness	1220	How likely are you to doze off or fall asleep during the daytime when you don't mean to? (e.g. when working, reading or driving)	Never/rarely; Sometimes;	Often; All the Time;

Table S2. Code lists used in the UK Biobank study to identify CKD disease cases

ICD-10
chronic nephritic syndrome: N03
isolated proteinuria with specified morphological lesion: N06
glomerular disorders in diseases classified elsewhere: N08
chronic tubulo-interstitial nephritis: N11
tubulo-interstitial nephritis, not specified as acute or chronic: N12
obstructive and reflux uropathy: N13
drug- and heavy-metal-induced tubulo-interstitial and tubular conditions: N14
other renal tubulo-interstitial diseases: N15
renal tubulo-interstitial disorders in diseases classified elsewhere: N16
chronic renal failure: N18
unspecified renal failure: N19
calculus of kidney and ureter: N20
calculus of lower urinary tract: N21
eGFR:< 60 mL/min per 1.73 m^2
UACR:> 30 mg/g

Abbreviations: CKD = chronic kidney disease; eGFR = estimated glomerular filtration rate; UACR = urinary albumin - creatinine ratio

Table S3. The numbers and percentages of participants with missing covariates.

Variables	N(%)
Education	2478 (0.68)
Ethnic background	922 (0.25)
Diabetes	21465 (5.93)
BMI	1567 (0.43)
Hypertension	22411 (6.19)
Smoking	890 (0.25)
Alcohol	160 (0.04)
Physical activity	6681 (1.85)

Abbreviations: BMI = body mass index.

Table S4. Subgroup analysis

Subgroup	Dietary zinc Supplementation		*p*-interaction	Sleep pattern			*p*-interaction
	Non-Zn Supp Group	Zn Supp Group		Poor	Moderate	Healthy	
Age			0.276				<0.001
<65	reference	0.93 (0.85–1.02)		reference	0.86 (0.81–0.92)	0.77 (0.72–0.83)	
≥65	reference	0.85 (0.75–0.97)		reference	0.92 (0.83–1.03)	0.91 (0.81–1.02)	
Sex			0.836				<0.001
Female	reference	0.93 (0.85–1.03)		reference	0.89 (0.81–0.97)	0.79 (0.73–0.87)	
Male	reference	0.90 (0.81–1.01)		reference	0.83 (0.77–0.90)	0.81 (0.75–0.88)	
Education			0.247				0.202
Higher degree	reference	0.96 (0.84–1.09)		reference	0.89 (0.78–1.01)	0.86 (0.76–0.98)	
Any school degree	reference	0.91 (0.81–1.03)		reference	0.84 (0.77–0.93)	0.76 (0.69–0.84)	
Vocational qualifications	reference	0.97 (0.80–1.19)		reference	0.84 (0.72–0.97)	0.75 (0.64–0.88)	

Ethnic background			0.363				0.516
Non-White	reference	1.04 (0.82–1.30)		reference	0.92 (0.75–1.13)	0.81 (0.65–1.00)	
White	reference	0.91 (0.84–0.98)		reference	0.85 (0.80–0.90)	0.80 (0.75–0.85)	
BMI			0.054				0.075
< 18.5	reference	1.95 (0.64–5.92)		reference	1.90 (0.25–14.29)	1.53 (0.20–11.79)	
18.5 - 25	reference	0.83 (0.72–0.97)		reference	0.75 (0.65–0.87)	0.67 (0.58–0.78)	
25 - 30	reference	0.91 (0.81–1.02)		reference	0.93 (0.85–1.03)	0.86 (0.78–0.95)	
⩾ 30	reference	1.01 (0.89–1.14)					
Hypertension			0.058				0.535
No	reference	0.92 (0.81–1.05)		reference	0.82 (0.74–0.92)	0.74 (0.66–0.83)	
Yes	reference	0.90 (0.82–0.98)		reference	0.88 (0.82–0.94)	0.83 (0.77–0.89)	
Diabetes			0.895				0.032
No	reference	0.91 (0.84–0.99)		reference	0.84 (0.79–0.90)	0.78 (0.73–0.83)	
Yes	reference	0.94 (0.76–1.16)		reference	0.92 (0.81–1.05)	0.90 (0.79–1.04)	

Notes: The “Non-Zn Supp Group” includes participants who have not used dietary zinc supplementation. The “Zn Supp Group” includes

participants who have used dietary zinc supplementation. Abbreviations: BMI = body mass index.

Table S5. Association between dietary zinc supplementation, sleep patterns, and CKD risk in individuals followed for more than two years.

Subgroup	Model 1		Model 2		Model 3	
	HR(95%CI)	*p*-value	HR(95%CI)	*p* -value	HR(95%CI)	*p* -value
Zn Suppl.						
No	1.00 (reference)		1.00 (reference)		1.00 (reference)	
Yes	0.85 (0.78–0.91)	<0.001	0.92 (0.86–1.00)	0.044	0.93 (0.86–1.00)	0.056
Sleep pattern						
Poor	1.00 (reference)		1.00 (reference)		1.00 (reference)	
Moderate	0.72 (0.68–0.77)	<0.001	0.83 (0.78–0.88)	<0.001	0.85 (0.80–0.91)	<0.001
Healthy	0.61 (0.58–0.65)	<0.001	0.77 (0.72–0.82)	<0.001	0.80 (0.75–0.85)	<0.001
P for trend		<0.001		<0.001		<0.001

Model 1: adjusted for age (continuous)), sex (male, or female). Model 2: further adjusted for race (white, or non-white), BMI (underweight, normal weight, overweight, or obese), education level (higher degree, any school degree, vocational qualifications, or none of the above), TDI, drinking status (never drinking, former drinking, current drinking), smoking status (never smoker, former smoker, or current smoker), physical activity (low, moderate, or high), vitamin supplementation (yes or no), other mineral supplementation (yes or no), diet score. Model 3: further adjusted for hypertension (yes, or no), diabetes (yes, or no). Abbreviations: Zn Suppl.= zinc supplementation; TDI = Townsend deprivation index; BMI = body mass index.

Table S6. Hazard ratios of dietary zinc supplementation and CKD risk stratified by sleep patterns among individuals followed for more than two years.

Zn Suppl.	Sleep pattern (HR, 95% CI)			*p* for interaction
	Poor sleep	Moderate sleep	Healthy sleep	
No	1.00 (reference)	1.00 (reference)	1.00 (reference)	0.018
Yes	0.69 (0.48–0.98)	0.90 (0.82–0.99)	1.02 (0.89–1.16)	

Adjusted for age (continuous)), sex (male, or female), race (white, or non-white), BMI (underweight, normal weight, overweight, or obese), education level (higher degree, any school degree, vocational qualifications, or none of the above), TDI, drinking status (never drinking, former drinking, current drinking), smoking status (never smoker, former smoker, or current smoker), physical activity (low, moderate, or high), diet score, vitamin supplementation (yes or no), other mineral supplementation (yes or no), hypertension (yes, or no) and diabetes (yes, or no). Abbreviations: Zn Suppl.= zinc supplementation.

Table S7. Association between dietary zinc supplementation, sleep patterns, and CKD risk among participants with and without CKD in a 1:4 propensity score matched cohort.

Subgroup	Model 1		Model 2		Model 3	
	HR(95%CI)	*p*-value	HR(95%CI)	*p*-value	HR(95%CI)	*p*-value
Zn Suppl.						
No	1.00 (reference)		1.00 (reference)		1.00 (reference)	
Yes	0.83 (0.77–0.89)	<0.001	0.90 (0.84–0.97)	0.006	0.91 (0.85–0.98)	0.010
Sleep pattern						
Poor	1.00 (reference)		1.00 (reference)		1.00 (reference)	
Moderate	0.73 (0.69–0.77)	<0.001	0.83 (0.78–0.88)	<0.001	0.85 (0.80–0.90)	<0.001
Healthy	0.63 (0.59–0.66)	<0.001	0.77 (0.73–0.82)	<0.001	0.80 (0.75–0.85)	<0.001
p for trend		<0.001		<0.001		<0.001

Model 1: adjusted for age (continuous)), sex (male, or female). Model 2: further adjusted for race (white, or non-white), BMI (underweight, normal weight, overweight, or obese), education level (higher degree, any school degree, vocational qualifications, or none of the above), TDI, drinking status (never drinking, former drinking, current drinking), smoking status (never smoker, former smoker, or current smoker), physical activity (low, moderate, or high), vitamin supplementation (yes or no), other mineral supplementation (yes or no), vitamin supplementation (yes or no), other mineral supplementation (yes or no), diet score. Model 3: further adjusted for hypertension (yes, or no), diabetes (yes, or no). Abbreviations: Zn Suppl.= zinc supplementation; TDI = Townsend deprivation index; BMI = body mass index.

Table S8. Hazard ratios of dietary zinc supplementation on CKD risk stratified by sleep pattern using a 1:4 propensity score matching approach.

Zn Suppl.	Sleep pattern (HR, 95% CI)			*p* for interaction
	Poor sleep	Moderate sleep	Healthy sleep	
No	1.00 (reference)	1.00 (reference)	1.00 (reference)	0.014
Yes	0.76 (0.54–1.05)	0.87 (0.79–0.95)	1.01 (0.89–1.14)	

Adjusted for age (continuous)), sex (male, or female), race (white, or non-white), BMI (underweight, normal weight, overweight, or obese), education level (higher degree, any school degree, vocational qualifications, or none of the above), TDI, drinking status (never drinking, former drinking, current drinking), smoking status (never smoker, former smoker, or current smoker), physical activity (low, moderate, or high), vitamin supplementation (yes or no), other mineral supplementation (yes or no), diet score, hypertension (yes, or no) and diabetes (yes, or no). Abbreviations: Zn Suppl.= zinc supplementation.

Table S9. Association between dietary zinc supplementation, sleep patterns, and CKD risk using disease code N18.

Subgroup	Model 1		Model 2		Model 3	
	HR(95%CI)	*p*-value	HR(95%CI)	*p*-value	HR(95%CI)	*p*-value
Zn Suppl.						
No	1.00 (reference)		1.00 (reference)		1.00 (reference)	
Yes	0.77 (0.70–0.85)	<0.001	0.87 (0.79–0.95)	<0.001	0.87 (0.79–0.96)	0.004
Sleep pattern						
Poor	1.00 (reference)		1.00 (reference)		1.00 (reference)	
Moderate	0.69 (0.64–0.74)	<0.001	0.81 (0.75–0.87)	<0.001	0.84 (0.78–0.90)	<0.001
Healthy	0.58 (0.54–0.62)	<0.001	0.75 (0.70–0.81)	<0.001	0.80 (0.74–0.86)	<0.001
p for trend		<0.001		<0.001		<0.001

Model 1: adjusted for age (continuous)), sex (male, or female). Model 2: further adjusted for race (white, or non-white), BMI (underweight, normal weight, overweight, or obese), education level (higher degree, any school degree, vocational qualifications, or none of the above), TDI, drinking status (never drinking, former drinking, current drinking), smoking status (never smoker, former smoker, or current smoker), physical activity (low, moderate, or high), vitamin supplementation (yes or no), other mineral supplementation (yes or no), diet score. Model 3: further adjusted for hypertension (yes, or no), diabetes (yes, or no). Abbreviations: Zn Suppl.= zinc supplementation; TDI = Townsend deprivation index; BMI = body mass index.

Table S10. Hazard ratios of dietary zinc supplement use on CKD risk, stratified by sleep pattern, employing disease code N18 exclusively.

Zn Suppl.	Sleep pattern (HR, 95% CI)			*p* for interaction
	Poor sleep	Moderate sleep	Healthy sleep	
No	1.00 (reference)	1.00 (reference)	1.00 (reference)	0.560
Yes	0.84 (0.58–1.24)	0.86 (0.76–0.97)	0.89 (0.76–1.06)	

Adjusted for age (continuous)), sex (male, or female), race (white, or non-white), BMI (underweight, normal weight, overweight, or obese), education level (higher degree, any school degree, vocational qualifications, or none of the above), TDI, drinking status (never drinking, former drinking, current drinking), smoking status (never smoker, former smoker, or current smoker), physical activity (low, moderate, or high), vitamin supplementation (yes or no), other mineral supplementation (yes or no), diet score, hypertension (yes, or no) and diabetes (yes, or no). Abbreviations: Zn Suppl.= zinc supplementation.

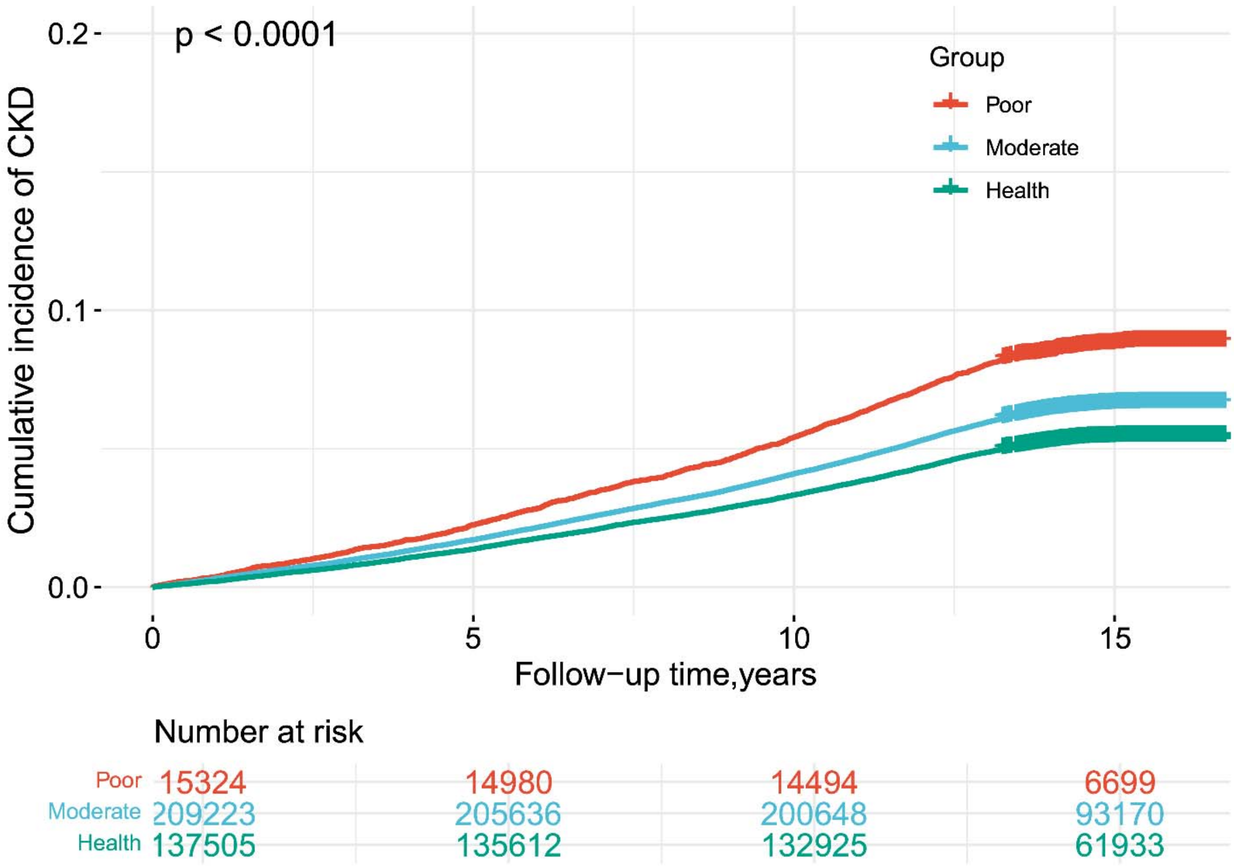

Figure S1. Cumulative incidence of CKD stratified by sleep patterns.

Abbreviations: Poor: Poor sleep pattern. Moderate: Moderate sleep pattern. Health: Health sleep pattern.
